# Effective therapeutic strategies against *Pseudomonas aeruginosa* and *Burkholderia Cepacia* complex infections

**DOI:** 10.1038/s41598-025-26712-8

**Published:** 2025-11-23

**Authors:** Noura A. M. Helmy, Ahmed F. Basyony, Sally T. K. Tohamy, Samar A. Zaki

**Affiliations:** 1 Department of Microbiology and Immunology, Faculty of Pharmacy, Egyptian Russian University, Badr City, Cairo Egypt; 2 Department of Laboratory Sciences, College of Pharmacy, University of Kut, Wasit, 52001 Iraq; 3 Department of Microbiology and Immunology, Faculty of Pharmacy (Girls), Al- Azhar University, Cairo, Egypt

**Keywords:** *Burkholderia Cepacia* complex, Pseudomonas aeruginosa, VITEK 2, *RecA* gene, Resistance, Antimicrobial combinations, Biological techniques, Microbiology, Molecular biology

## Abstract

*Pseudomonas aeruginosa* and *Burkholderia cepacia* complex (Bcc) can cause serious nosocomial infections. The reported misidentification of Bcc as *P. aeruginosa*, besides the contrasting susceptibility patterns between these two pathogens, underscores the importance of accurate identification and the need to explore therapeutic approaches that could eradicate them both. Our study aimed to evaluate the susceptibilities of both pathogens to different antibiotics and test the effectiveness of different antimicrobial combinations. In addition, we compared conventional phenotypic and automated identification methods using the VITEK 2 system with PCR amplification and sequencing of *recA* gene to differentiate between *P. aeruginosa* and Bcc. Amplification by PCR of the *recA* gene showed high specificity in the identification of Bcc in comparison with conventional and automated phenotypic methods. Significant differences in susceptibility were observed with *P. aeruginosa* isolates having more susceptibility to colistin and ceftazidime, while *Burkholderia* isolates were more susceptible to cotrimoxazole and minocycline. Both *P. aeruginosa* and *Burkholderia* isolates tested were more susceptible to N-Acetyl-L-Cysteine (NALC) plus meropenem or levofloxacin, followed by gentamicin plus piperacillin/tazobactam. Synergistic effect of NALC plus meropenem or levofloxacin may help in the eradication of both pathogens to overcome the diagnostic challenges posed by the possible misidentification using conventional identification methods.

**Supplementary Information:**

The online version contains supplementary material available at 10.1038/s41598-025-26712-8.

## Introduction

*Burkholderia cepacia* complex (Bcc) and *Pseudomonas aeruginosa* are opportunistic microorganisms that have been isolated from different sources, including industrial settings, water, soil, and plants^1^. Exploring the NCBI whole taxonomy using the life map interactive tool revealed that Bcc currently contains 29 different species^2^. It is well-recognized that it can cause severe respiratory tract infections in cystic fibrosis (CF) patients^3^, and has become recognized as a significant human pathogen in hospitalized and immunocompromised patients who can develop the Bcc infection after dealing with contaminated devices. It can result in infections such as bacteremia, septic arthritis, and peritonitis in addition to urinary tract and respiratory tract infections^4^. On the other hand, *P. aeruginosa* is the most common cause of long-lasting infections in CF patients, accounting for 80% of cases in adults suffering from this condition^5^. *Pseudomonas aeruginosa* can infect other vulnerable patients, such as those with pulmonary disorders, intubated, or have persistent bladder catheters, thus typically causing infections in the urinary and airway systems^6^. It is one of the causative agents of nosocomial infections resulting in pneumonia, sepsis, and meningoencephalitis. It frequently colonizes medical devices as nebulizers, catheters, and humidifiers^7^, and is also a significant pathogen in diabetic foot disease, burns, and wounds^8^.

Bacteria of Bcc have frequently been mistaken for other Gram-negative non-fermentative bacilli, particularly *Pseudomonas* species^9–11^. This clarifies the reason why there are not many reports regarding Bcc infections in Egypt and other nations^12^. It is difficult to distinguish between distinct species within the Bcc as well as between these species and other closely related taxa^10^. Studies have demonstrated that phenotypic tests are not appropriate for the detection of Bcc species, and they cannot provide reliable findings when using manual or automated systems like VITEK 2, VITEK MS, and Phoenix^13^. This can be attributed to factors such as the phenotypic similarity between the different species of Bcc^14^. The Bcc consists of different genetically distinct species that exhibit highly similar biochemical profiles^15^. Also, database limitation could mainly be attributed to limitations encountered with automated phenotypic identification systems^16^. To identify the bacteria in this complex, molecular techniques are usually employed, typically involving PCR and analysis of target genes including 16 S rRNA, *recA*, and *hisA* sequences^13^. Sequencing of *recA*, a housekeeping gene, was found to be an effective technique for identifying the species^17^. For bacterial identification, the sequence analysis of 16 S and 23 S rRNA are frequently used. However, for this complex, this approach is able to identify the genus but cannot differentiate to the species level^14^. The conventional and automated phenotypic identification are the routine identification methods in healthcare facilities, while molecular techniques are not the regularly employed methods for identification especially in developing countries with poor infrastructure^18^ where misidentification usually occurs^9^ so there was a need to explore therapeutic approaches that could ensure coverage of both pathogens.

Multidrug-resistant (MDR), extensively-drug-resistant (XDR), and pan-drug-resistant (PDR) terms are being used to characterize different resistance patterns found in antimicrobial-resistant, healthcare-associated microorganisms. MDR was defined as acquired non-susceptibility to at least one agent in three or more antimicrobial categories, XDR was defined as non-susceptibility to at least one agent in all but two or fewer antimicrobial categories (i.e., bacterial isolates remain susceptible to only one or two categories), and PDR was defined as non-susceptibility to all agents in all antimicrobial categories^19^.

Infections caused by the Bcc exhibit difficulty in treatment due to the high incidence of multidrug resistance^20^. Species in the Bcc are considered as highly resistant microorganisms, due to their extremely high levels of innate resistance to a wide variety of antimicrobial compounds^21^. Bcc has an intrinsically different drug susceptibility pattern from *P. aeruginosa*. It has intrinsic resistance to antimicrobial agents, including first and second-generation cephalosporins, aminoglycosides, polymyxins, and antipseudomonal penicillins^4^. Whereas, in *P. aeruginosa* infections, these different categories of antimicrobial agents are frequently used^4,12,22^.

In light of the increasing resistance to antimicrobial agents and the scarcity of alternative treatment options, a significant amount of research has been conducted to identify possible therapeutic adjuvants that can potentially enhance the efficiency of antimicrobial agents^23^. Among them is N-Acetyl-L-Cysteine (NALC), an endogenous glutathione precursor with mucolytic, anti-inflammatory, and antioxidant properties, that is currently used as an inhaled mucolytic in clinical practice to manage the excessively thick mucus produced by patients with CF or obstructive pulmonary diseases^24^. An increasing amount of research indicates that NALC possesses antibacterial and antibiofilm activities against serious respiratory pathogens, including *P. aeruginosa*^25^, Bcc and *Stenotrophomonas maltophilia*^26^.

Our study aimed to investigate different antimicrobial agents, tested in dual and triple combinations, with the objective of identifying effective combinations that are capable of eradicating both pathogens to overcome the diagnostic challenges posed by the misidentification using conventional phenotypic methods.

## Materials and methods

### Standard strains

*Burkholderia cenocepacia* (ATCC BAA/245) was used as a control in the sensitivity test and positive control in polymerase chain reaction (PCR) for the detection of the *recA* gene. *Pseudomonas aeruginosa* (PAO1) was used as a control in the sensitivity test and negative control in PCR for the detection of the *recA* gene. *Escherichia coli* (ATCC 25922) was used as a control in the sensitivity test.

### Collection of specimens

Clinical specimens were collected by specialized clinicians from different hospitals in Cairo, Egypt. These clinical specimens were collected from different infection sites, including blood, urine, sputum, wound infection, surgical drains, central lines, and chest tubes.

This study involves the use of clinical specimens that were collected by specialized clinicians as part of their routine diagnostic procedures, with no direct interaction with patients. Institutional review board (the Ethics Committee of the Faculty of Pharmacy (Girls) at Al-Azhar University, Egypt, under approval number [245]) approved a waiver of informed consent due to minimal risk and impracticability of obtaining consent.

### Isolation, and purification

Directly upon receiving, all specimens, except for blood specimens, were inoculated on blood, chocolate, and MacConkey agar media (Oxoid, United Kingdom) and the plates were incubated inverted under aerobic conditions at 35 °C (± 2) for 16 to 24 h. Blood specimens were directly injected into blood culture media (Egyptian diagnostic media, Egypt), they were incubated at 37 °C, and examined daily for up to 14 days by looking for turbidity. When the blood culture was revealed positive, 1 mL of the inoculated broth was inoculated on blood, chocolate, and MacConkey agar. If the blood culture was revealed negative after 14 days, 1 mL was also inoculated on blood agar for confirmation of the negative result before discarding. Growth obtained on blood and chocolate agar was purified by streaking on the surface of MacConkey agar media so that well-isolated colonies were obtained.

### Phenotypic identification

#### Conventional methods

A total of 110 cultured clinical isolates were included in the study according to their Gram staining, oxidase test results (carried out using oxidase reagent discs (Oxoid, United Kingdom)), and catalase test results (carried out using 3% hydrogen peroxide). The isolates were then categorized according to growth on cetrimide (HiMedia, India), and growth on *Burkholderia cepacia* selective agar (BCSA) (1% sucrose and 1% lactose in yeast extract and casein-enriched base with 2.5 µg vancomycin/mL, 10 µg gentamicin/mL, and 600 IU polymyxin B/mL)^10^.

#### Automated method

Automated identification of the 110 previously cultured clinical isolates was performed by the VITEK^®^ 2 System, version 9.02 software (bioMérieux, USA) in El-Demerdash hospital using the Gram-negative card^14^.

#### Intrinsic resistance

According to CLSI 2022, Bcc has intrinsic resistance to colistin in contrast to *P. aeruginosa*, while the latter has intrinsic resistance to tigecycline, cotrimoxazole, and chloramphenicol, which is in contrast to Bcc. The susceptibility of the standard *P. aeruginosa* (PAO1) and *B. cenocepacia* (BAA/245) was tested for chloramphenicol, tigecycline, and cotrimoxazole sensitivity using the Kirby-Bauer disk diffusion method^27^, while the susceptibility of both standards was tested for colistin using the broth microdilution method^28^.

### Genotypic identification

#### Chromosomal DNA extraction

Chromosomal DNA extraction was performed using Thermo Scientific GeneJET Genomic DNA Purification Kit according to the manufacturer`s specifications. The extracted DNA was validated qualitatively by mixing 5 µL of DNA with 1 µL of 6X DNA loading dye (Thermofisher Scientific, USA) and running it on an agarose gel (via gel electrophoresis). Quantification of the extraction was made using a NanoDrop 2000 spectrophotometer (Thermofisher Scientific, USA), and purity was checked by measuring the 260/280 absorbance ratio, with acceptable purity to be in the range of 1.8–1.9^29^. Then aliquots of 10 µL of the extracted DNA were preserved at – 20 °C.

#### Primers

PCR reactions were performed for amplification of the *recA* gene in Bcc using BCR1 (5′-TGACCGCCGAGAAGAGCAA-3′) as a forward primer and BCR2 (5′-CTCTTCTTCGTCCATCGCCTC-3′) as a reverse primer with an expected product size of 1043 bp^15,30^. Primers were synthesized by Invitrogen custom primers, supplied by Thermofisher Scientific, USA.

#### Amplification of the *RecA* gene using PCR

The composition of the PCR mixtures was described in Supplementary Table 1. The condition of PCR cycles included 1 cycle of initial denaturation at 98 °C for 5 min, 35 cycles of denaturation at 98 °C for 10 s, annealing at 63 °C for 20 s, then elongation at 72 °C for 20 s. The final step was 1 cycle of final elongation at 72 °C for 5 min. The amplified PCR products were detected using agarose gel electrophoresis. The PCR products were loaded on an agarose gel at 1% with ethidium bromide that was added to the warm gel at a 0.5 µg/mL final concentration. The DNA ladder was also loaded into the assigned well of the gel. The electric current was applied at 4–10 V/cm. The amplified DNA was visualized by UV transilluminator (Acculab, France).

#### Sequencing of *RecA* gene

**Fig. 1:**
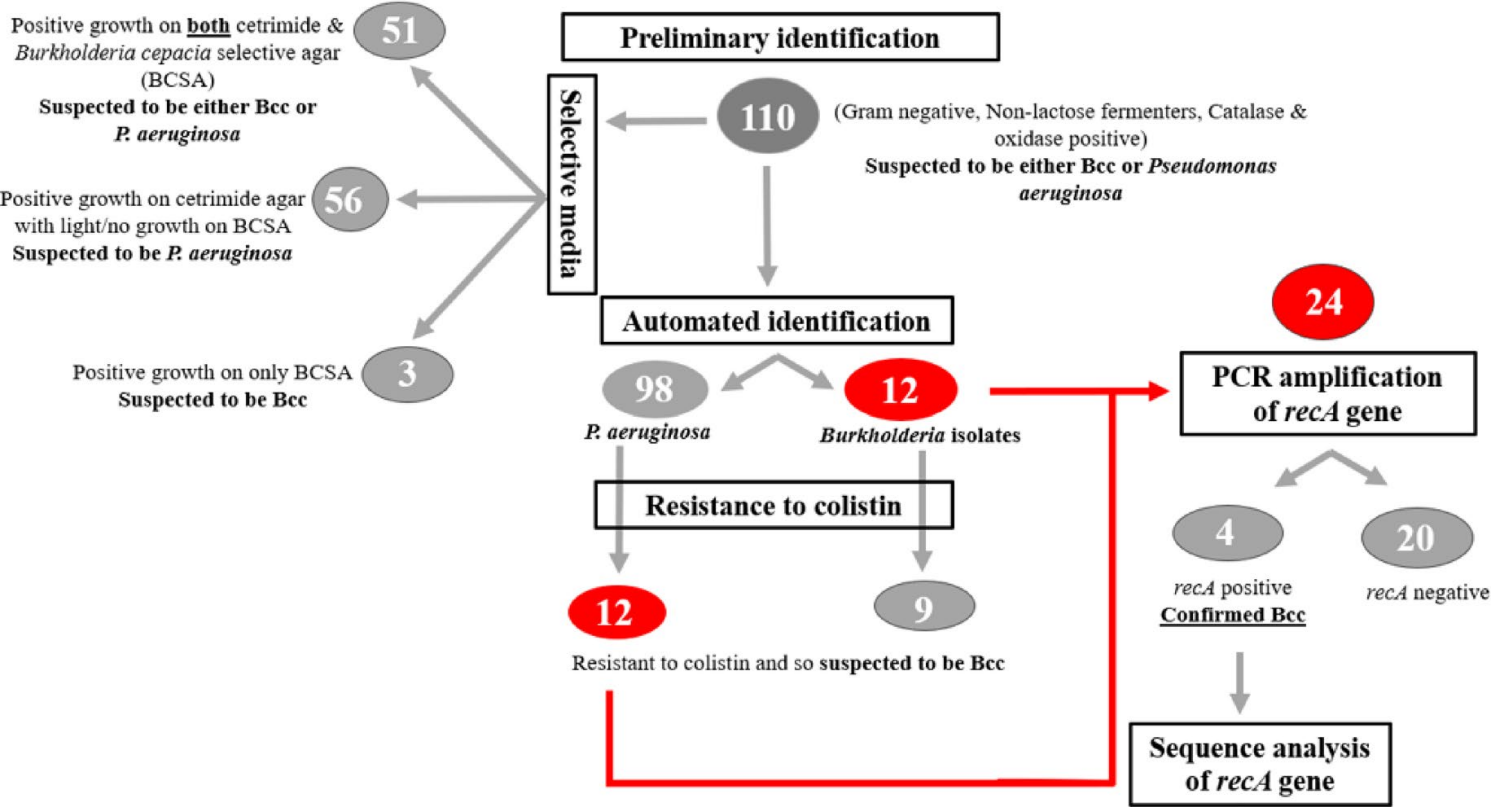
Flow chart of the study design illustrating identification steps.

The PCR products were sent to Sigma Scientific Services Company, Egypt; prior to sequencing, they were treated with the DNA Clean and Concentrator-25 purification kit (ZYMO RESEARCH, USA). Bidirectional sequencing was carried out at GATC Company, Germany, using the API 3730 × 1 DNA sequencer. The obtained *recA* gene sequence of the selected isolates was compared with those in the GenBank database using the nucleotide BLAST tool (http://blast.ncbi.nlm.nih.gov/Blast.cgi) for the identification of the test isolates using the parameters of percentage identity more than 95% and E-value equal to zero then the phylogenetic tree was assembled for each of the tested isolates’ sequences using the neighbor-joining algorithm in the nucleotide BLAST tool. In addition, the obtained *recA* gene sequences were analyzed using PubMLST database (https://pubmlst.org/). Then, multiple sequence alignment was performed using the MUSCLE program^31^ for the 4 *recA* gene sequences of the previously identified *Burkholderia* isolates. A phylogenetic tree was constructed using the neighbor-joining algorithm, including all tested isolates along with one or two representative sequences for each *Burkholderia* species in a single phylogenetic tree. Multiple sequence alignment and evolutionary analysis were conducted in MEGA11^32^. A flow chart of the study design for all identification steps was illustrated in Fig. 1.

### Antimicrobial susceptibility test

#### Kirby-Bauer disc diffusion susceptibility test

Kirby-Bauer disc diffusion method^33^ was performed using the following antimicrobial discs: gentamicin (CN, 10 µg), meropenem (MRP, 10 µg), ceftazidime (CAZ, 30 µg), minocycline (MI, 30 µg), and cotrimoxazole (COT, 25 µg). All discs were purchased from Oxoid, England. CLSI breakpoints for antimicrobial susceptibility testing of *P. aeruginosa* were used for interpreting the results of meropenem, ceftazidime, and gentamicin against *P. aeruginosa* isolates, while for minocycline and cotrimoxazole breakpoints of Bcc were used. Breakpoints of Bcc were used to interpret the results of minocycline, meropenem, cotrimoxazole, and ceftazidime against *Burkholderia* species isolates^34^, while for gentamicin, the breakpoints of *P. aeruginosa* were used^35,36^.

#### Determination of the minimum inhibitory concentration (MIC)

The MICs of colistin (CST), levofloxacin (LVX), and piperacillin/tazobactam (TZP) were determined using the broth microdilution method, according to CLSI guidelines^28^. CLSI breakpoints for antimicrobial susceptibility testing of *P. aeruginosa* were used to interpret the MIC results of *P. aeruginosa* isolates. Concerning *Burkholderia* species isolates, MICs for colistin and piperacillin/tazobactam were also interpreted according to the CLSI breakpoints for antimicrobial susceptibility testing of *P. aeruginosa*^35,36^ while levofloxacin MICs were interpreted according to the CLSI breakpoints for antimicrobial susceptibility testing of Bcc^34^.

### Detection of multidrug-resistant bacteria

In the current study, eight antimicrobial agents representing eight different categories were tested against the provided clinical isolates, so only MDR bacteria could be detected. The tested categories were beta-lactam combinations (piperacillin-tazobactam), cephalosporin antibiotics (ceftazidime), carbapenems (meropenem), tetracyclines (minocycline), fluoroquinolones (levofloxacin), folate antagonists (trimethoprim-sulfamethoxazole), aminoglycosides (gentamicin), and lipopeptides (colistin). *Burkholderia species* have intrinsic resistance to lipopeptides (colistin)^37^ so, resistance to this antimicrobial agent was neglected in the determination of MDR isolates. On the other hand, *Pseudomonas aeruginosa* has intrinsic resistance to tetracyclines (minocycline) and folate antagonists (trimethoprim-sulfamethoxazole)^34^, so resistance to these antimicrobial agents was neglected in the determination of MDR isolates.

### Determination of the effect of the antimicrobial combinations

#### Double disc synergy assay

Double disc synergy was tested on both standards (BAA/245 and PAO1) for minocycline, meropenem, cotrimoxazole, ceftazidime, gentamicin, and levofloxacin as a preliminary method for the detection of any possible synergism. Inoculum prepared as in the Kirby-Bauer disc diffusion susceptibility test^33^. The dried surface of the Mueller-Hinton agar plate was inoculated then **t**he appropriate antimicrobial discs were placed on the surface of the agar to be separated apart by the sum of radii of the inhibition zone of each drug separately and incubated at 37 °C. Synergy was indicated by bridging the zone of inhibition^38^.

#### Checkerboard assay

The effect of the promising antimicrobial combinations was tested using a checkerboard assay on both standards (BAA/245 and PAO1). Antimicrobial combinations tested were CN + MRP, COT + MRP, LVX + MRP, TZP + CN, NALC + MRP, and NALC + LVX. To determine the antimicrobial concentration range tested in the checkerboard assay, the MIC was first tested for each antimicrobial agent. Each well of the microtiter plate was filled with 100 µL aliquots of the double strength cation adjusted Mueller Hinton broth medium by multichannel pipette, proceeding by column from left to right or vice versa. Antimicrobial A was applied horizontally, and antimicrobial B was applied vertically to obtain mixtures of these compounds serially diluted. Subsequently, bacterial suspension equivalent to 0.5 McFarland standard (1 to 2 × 10^8^ CFU/mL) was diluted (1:150) and 100 µL inoculated, totaling 200 µL in each well^39^. Drugs A and B were examined as single agents, and the MIC for each of them was recorded. Each combination well was examined, and the results were recorded as growth or no growth for each well. For each combination interaction, the fractional inhibitory concentration (FIC) of each agent was calculated as follows:

$${\mathbf{FIC}}\;{\mathbf{of}}\;{\mathbf{agent}}\;{\mathbf{A}}{\text{ = MIC}}\;{\text{ of}}\;{\text{ agent }}\;{\text{A}}\;{\text{ in}}\;{\text{ combination/MIC}}\;{\text{ of }}\;{\text{agent}}\;{\text{ A}}\;{\text{ alone}}.$$

$${\mathbf{FIC }}\;{\mathbf{of }}\;{\mathbf{agent }}\;{\mathbf{B}}{\text{ = MIC }}\;{\text{of}}\;{\text{ agent}}\;{\text{ B}}\;{\text{ in}}\;{\text{ combination/MIC}}\;{\text{ of}}\;{\text{ agent}}\;{\text{ B }}\;{\text{alone}}.$$

The summation of the FIC (**∑**FIC) index for each combination was calculated as follows: **∑FIC = FIC of agent A + FIC of agent B.** The results were recorded as synergism, partial synergism, indifference, or antagonism if the **∑**FIC is ≤ 0.5, ranging between 0.5 and 1, ≥ 1 to < 4, or ≤ 4, respectively^40^.

#### Multiple-combination bactericidal test (MCBT)

Combinations of two antimicrobial agents were tested at physiologically achievable concentrations against both standards and 53 clinical isolates, 12 of them were *Burkholderia* species and 41 were MDR *P. aeruginosa* clinical isolates. Then, followed by testing the effect of three antimicrobial agents against the resistant isolates to the dual combinations. The working solutions of the antimicrobial agents used in the MCBT were prepared in double-strength cation-adjusted Mueller-Hinton broth at 10 times the final concentrations required in wells. These final concentrations corresponded to the resistance criteria. The antimicrobial agents were tested at the following fixed concentrations: MRP (16 and 8 µg/mL), TZP (128/4 µg/mL), CN (16 µg/mL), NALC (6250 µg/mL), LVX (8 and 4 µg/mL), and CST (4 µg/mL).

**Fig. 2:**
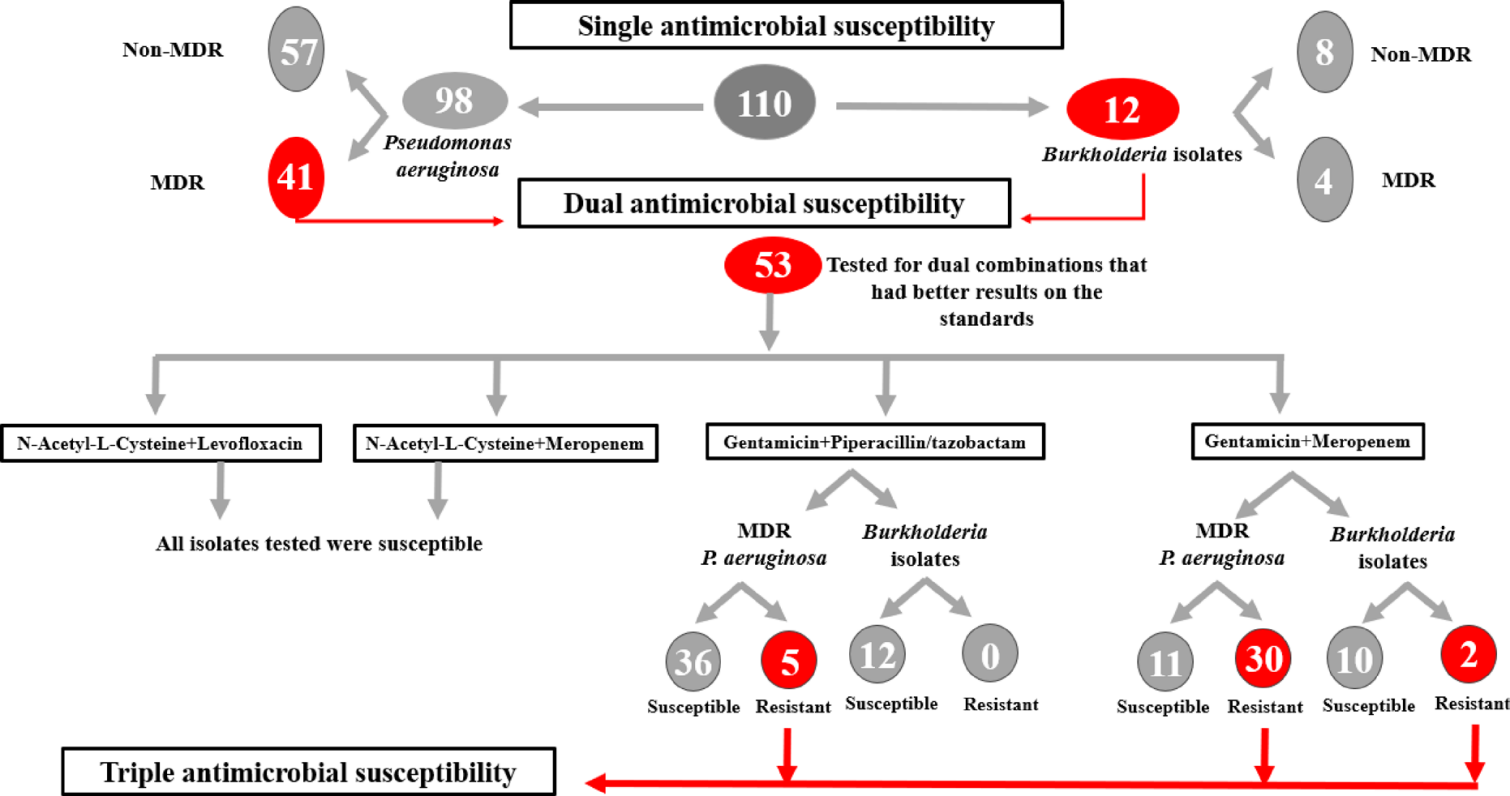
Flow chart of the study design illustrating antimicrobial susceptibility testing steps.

Combinations of two or three antimicrobial agents were placed in 96-well, round-bottomed microtiter plates, each in 10 µL volumes, into the appropriate wells. The necessary volume of double-strength cation-adjusted Mueller-Hinton broth was then added to the wells containing two antimicrobial agents so that all the wells had a volume of 30 µL. Subsequently, bacterial suspension equivalent to 0.5 McFarland standard (1 to 2 × 10^8^ CFU/mL) was diluted (1:100) and 70 µL inoculated. All plates included sterility and growth control wells (no inoculum and no antibiotic, respectively), then the plates were incubated at 35 °C for 48 h. At 24 and 48 h, the wells were examined for turbidity. The non-turbid well content was sub-cultured after 48 h by streaking 10 µL on 5% Columbia sheep blood agar plates, which were then incubated at 35 °C for 24 h. The combinations were considered synergistic if bactericidal activity of 99.9% killing was achieved^40–42^. A flow chart of the study design for steps of antimicrobial susceptibility testing was illustrated in Fig. 2.

### Statistical analysis

IBM SPSS^®^ Statistics version 26 (IBM^®^ Corp., Armonk, NY, USA) was used for the statistical analysis. Frequency and percentage were used to represent qualitative data. The relationship between qualitative variables was investigated using Pearson’s Chi-square test or Fisher’s exact test. All tests were two-tailed. A p-value less than 0.05 was considered to be significant.

## Results

### Collected bacterial isolates

A total number of 110 bacterial isolates were recovered from different clinical specimens, that were collected by specialized clinicians, according to the criteria of being Gram-negative, oxidase-positive, catalase-positive, and having positive growth on MacConkey agar as non-lactose fermenters. The specimens collected from different sites of infection, including blood (*n* = 13), urine (*n* = 39), sputum (*n* = 42), wound infection (*n* = 12), surgical drains (*n* = 1), central lines (*n* = 2), and chest tubes (*n* = 1).

### Phenotypic identification of the collected clinical isolates

#### Conventional methods

Concerning growth on BCSA, 54 isolates showed heavy growth and were so suspected to be Bcc, while 41 isolates were partially inhibited (indicated as light growth on the media after 3 days of incubation), and only 15 isolates showed negative growth. For cetrimide agar, 107 isolates showed positive growth and were so suspected to be *P. aeruginosa*, and only 3 isolates showed no growth.

#### Automated method

Automated identification of the collected clinical isolates using the VITEK 2 system revealed that 9 (8.2%) isolates were Bcc, 3 (2.7%) isolates were *Burkholderia* species, and 98 (89.1%) were *P. aeruginosa*.

#### Intrinsic resistance

The susceptibility of the standard strains PAO1 and BAA/245 was tested for chloramphenicol, tigecycline, cotrimoxazole, and colistin. The results showed that both standard strains were resistant to tigecycline. On the other hand, both standard strains were susceptible to cotrimoxazole. For chloramphenicol, BAA/245 was susceptible and PAO1 was intermediate-resistant, while for colistin, BAA/245 was resistant and PAO1 was susceptible. According to this finding, the results of susceptibility for colistin could be promising for differentiation between both microorganisms. Thus, the susceptibility was tested for all clinical isolates to colistin, and only 21 isolates were resistant (12 were identified by the automated method as *P. aeruginosa*, 2 as *Burkholderia* species, and 7 as Bcc).

### Genotypic identification

#### Amplification of *RecA* gene using PCR

Based on the results of the phenotypic identification using the automated method (VITEK 2) and the intrinsic resistance to colistin, a total of 24 clinical isolates in addition to the standards PAO1 (as negative control) and BAA/245 (as positive control) were chosen for testing the presence of the *recA* gene using PCR amplification technique. Out of the genotypically tested clinical isolates, 12 were identified by automated method (VITEK 2) as *Burkholderia* isolates, while the other 12 were identified as *P. aeruginosa*, which were colistin-resistant. The results revealed that an expected product of 1040 base pairs was detected for BAA/245 and 4 clinical isolates after agarose gel electrophoresis of the PCR amplified products. Ethidium bromide-stained gel electrophoresis for the tested clinical isolates` PCR products was illustrated in Supplementary Fig. 1.

#### Sequencing of *RecA* gene and construction of a phylogenetic tree

**Fig. 3:**
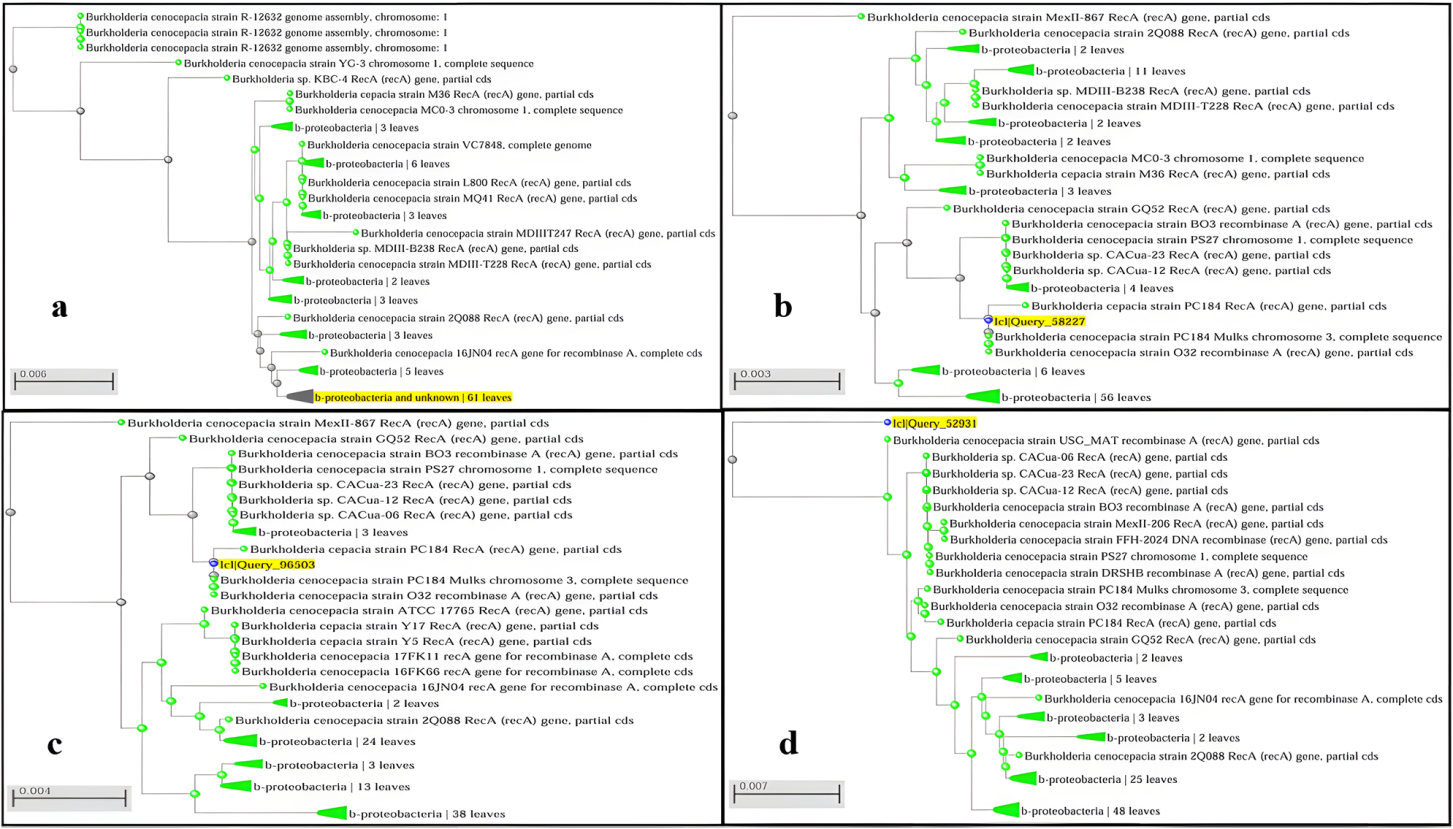
Phylogenetic tree for ***recA*** gene sequence of tested isolates using the neighbor-joining algorithm. *a: represents phylogenetic tree for *recA* gene sequence of Bur 5 isolate; **b**: represents phylogenetic tree for *recA* gene sequence of Bur 8 isolate; **c**: represents phylogenetic tree for *recA* gene sequence of Bur 9 isolate; **d**: represents phylogenetic tree for *recA* gene sequence of Bur 10 isolate.

**Fig. 4:**
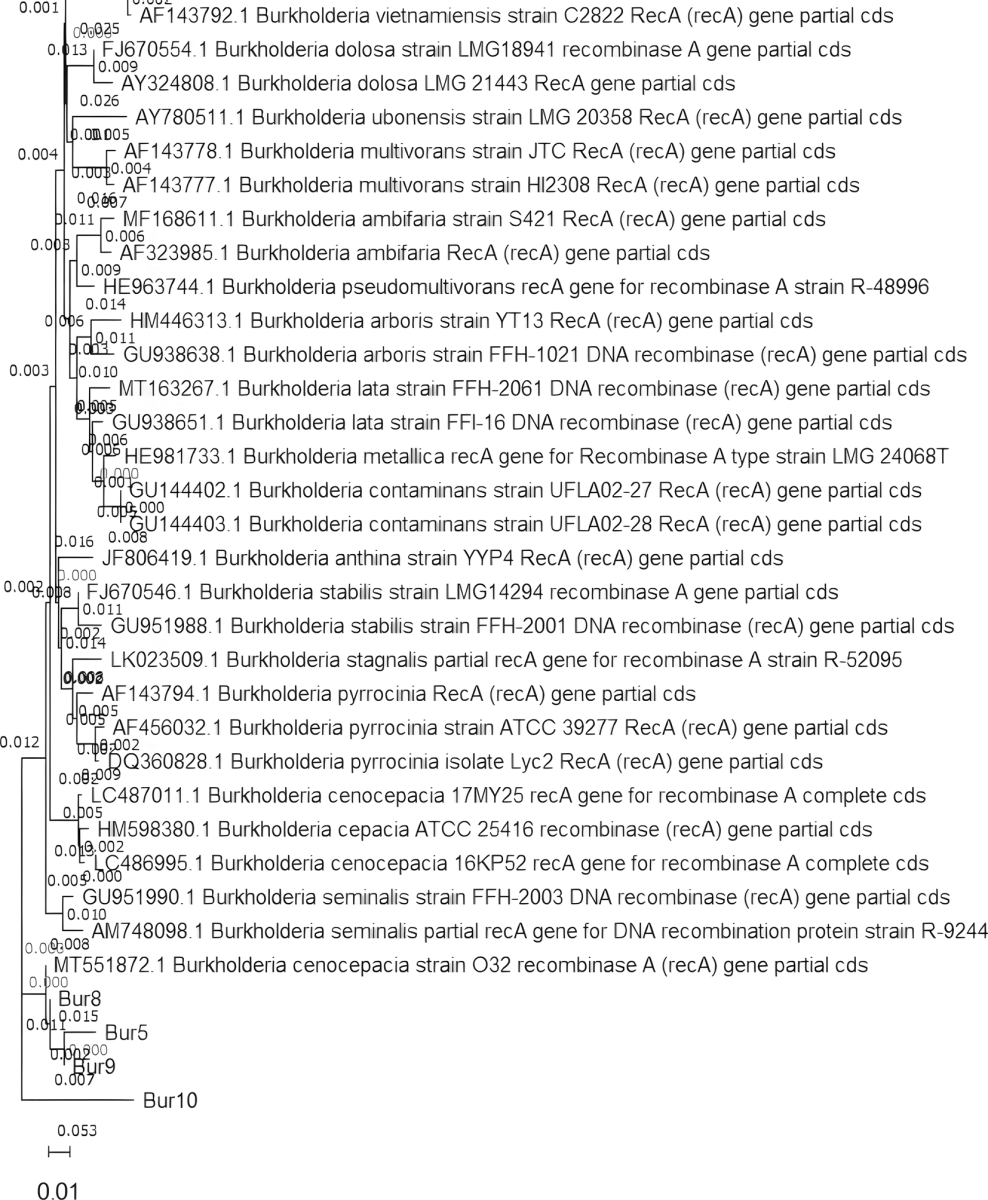
Evolutionary relationship for the 4 ***recA*** gene sequences of the identified *Burkholderia cenocepacia* clinical isolates (Bur5, Bur8, Bur9, and Bur10) along with representatives for each species of the *Burkholderia cepacia* complex. *The evolutionary relationship was inferred using the Neighbor-Joining method. The tree was drawn to scale, with branch lengths (next to the branches) in the same units as those of the evolutionary distances used to infer the phylogenetic tree. The evolutionary distances were computed using the Maximum Composite Likelihood method and are in the units of the number of base substitutions per site. This analysis involved 39 nucleotide sequences. Evolutionary analysis was conducted using MEGA11.

The obtained *recA* gene sequences for the amplified DNA product of the four clinical isolates that gave PCR bands of the expected size (Bur5, Bur8, Bur9, Bur10) were compared with those in the GenBank database using the nucleotide BLAST tool (http://blast.ncbi.nlm.nih.gov/Blast.cgi). The pairwise comparison revealed a percentage of identity more than 96% between each tested sequence and the nucleotide sequences obtained from the database after using the BLAST tool and all with an E-value equal to zero. The BLAST result of each clinical isolate sequence under investigation helped in the identification of the four isolates as *B. cenocepacia*. The phylogenetic trees for these sequences using the neighbor-joining algorithm with the selected isolates obtained using nucleotide BLAST and the NCBI database were assembled, and the results were illustrated in Fig. 3. On analysis of the *recA* gene sequences using PubMLST database, all sequences had a closest match to Bcc 13,370 (*recA*): allele 10. The evolutionary relationship for the 4 *recA* gene sequences of the identified *Burkholderia cenocepacia* clinical isolates, along with one or two representative sequences for each *Burkholderia* species, was assembled and illustrated as a single phylogenetic tree in Fig. 4.

### Antimicrobial susceptibility test

#### Kirby-Bauer disc diffusion susceptibility test

Results derived from the Kirby-Bauer disc diffusion susceptibility test showed that the majority of *Burkholderia* species, including the Bcc isolates, were susceptible to cotrimoxazole, with 11 (91.6%) isolates susceptible, followed by minocycline and meropenem, with 10 (83.3%) isolates susceptible, and gentamicin, with 4 (33.3%) isolates susceptible. None of the tested *Burkholderia* isolates were susceptible to ceftazidime.

**Fig. 5:**
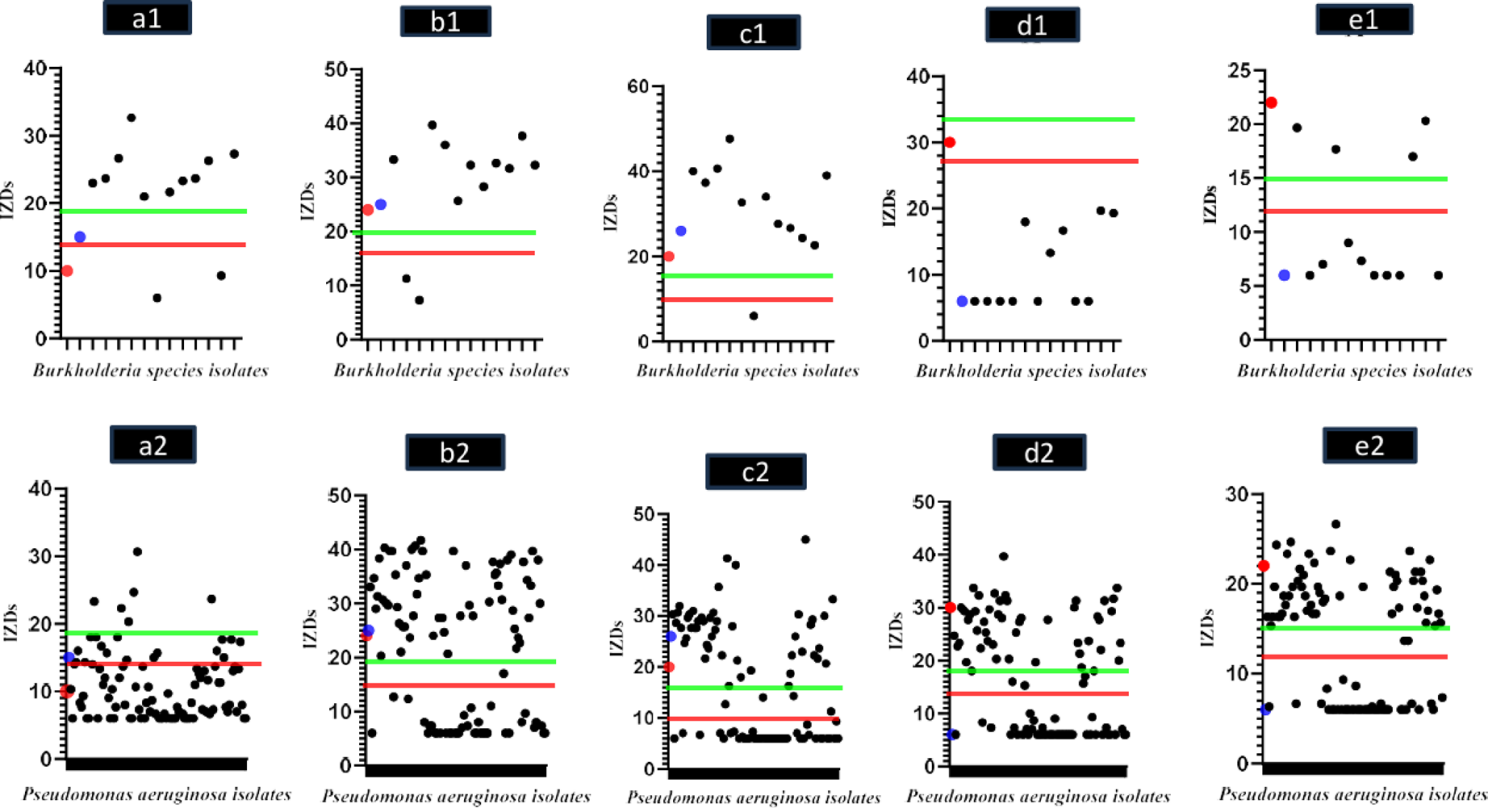
The inhibition zone diameters (IZDs) and susceptibility pattern of the clinical isolates. *a1. IZDs of minocycline against *Burkholderia* species isolates, **a2.** IZDs of minocycline against *P. aeruginosa* isolates; **b1.** IZDs of meropenem against *Burkholderia* species isolates, **b2.** IZDs of meropenem against *P. aeruginosa* isolates; **c1.** IZDs of cotrimoxazole against *Burkholderia* species isolates, **c2.** IZDs of cotrimoxazole against *P. aeruginosa* isolates; **d1.** IZDs of ceftazidime against *Burkholderia* species isolates, **d2.** IZDs of ceftazidime against *P. aeruginosa* isolates; **e1.** IZDs of gentamicin against *Burkholderia* species isolates, **e2.** IZDs of gentamicin against *P. aeruginosa* isolates. **Note that** the green line in the previous figures represents the lowest value of IZD for the isolates that can be considered susceptible, while the red line represents the highest value of IZD for the isolates that can be considered resistant. Dots in between green and red lines represent intermediate-resistant isolates. The red dot represents *P. aeruginosa* standard strain (PAO1) while the blue dot represents *Burkholderia cenocepacia* standard strain (BAA/245).

On the other hand, the majority of *P. aeruginosa* isolates were susceptible to meropenem, with 59 (60.2%) isolates susceptible, then gentamicin, with 55 (56.1%) isolates susceptible, ceftazidime, with 50 (51%) isolates susceptible, and cotrimoxazole, with 46 (46.9%) isolates susceptible while minocycline had the least activity, with only 6 (6.12%) isolates susceptible. The IZDs of the clinical isolates to different antimicrobial agents were summarized in Fig. 5.

#### Minimum inhibitory concentration test results

**Fig. 6:**
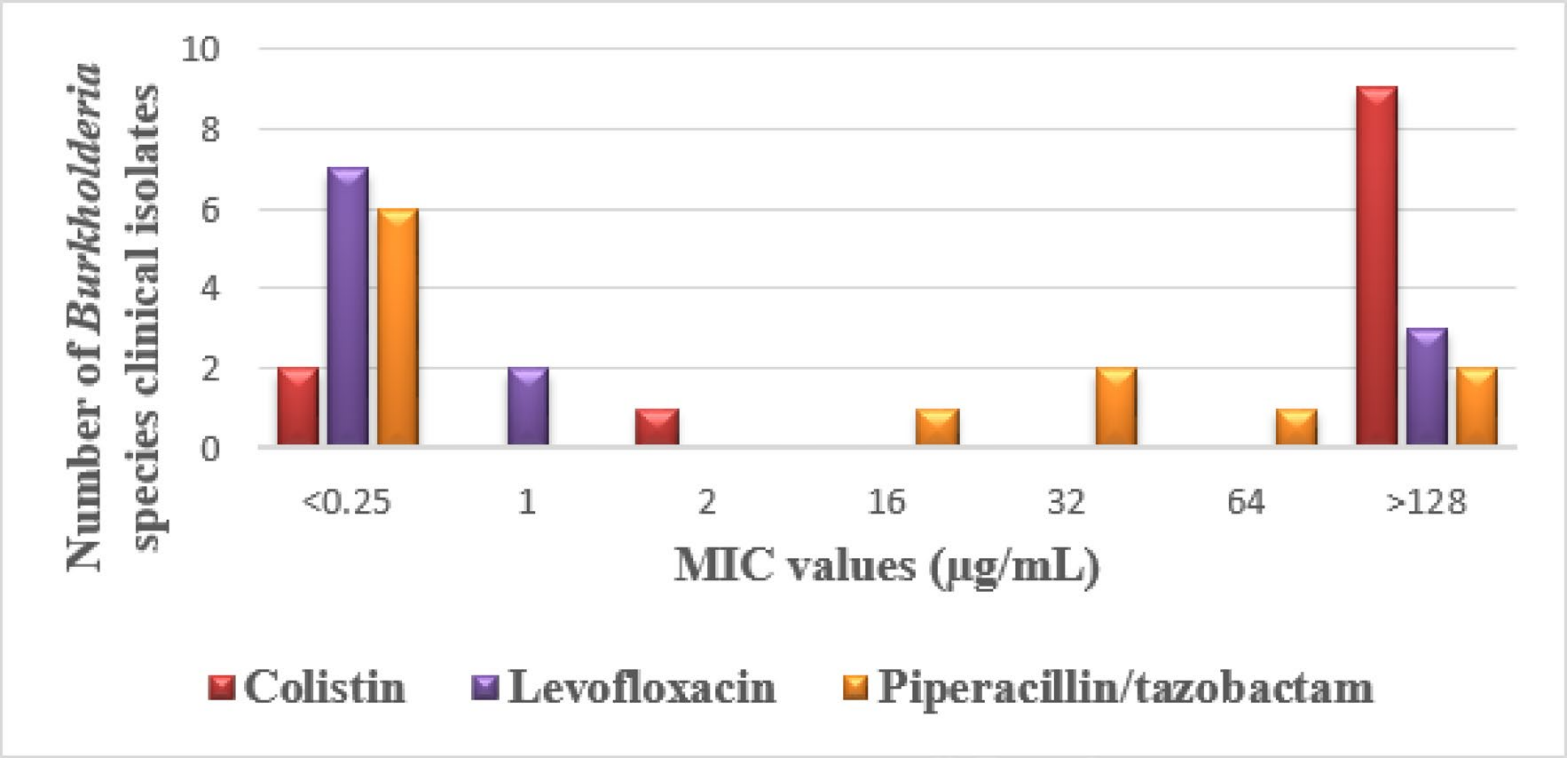
The minimum inhibitory concentration (MIC) values of the tested antimicrobial agents against the *Burkholderia* species clinical isolates.

**Fig. 7:**
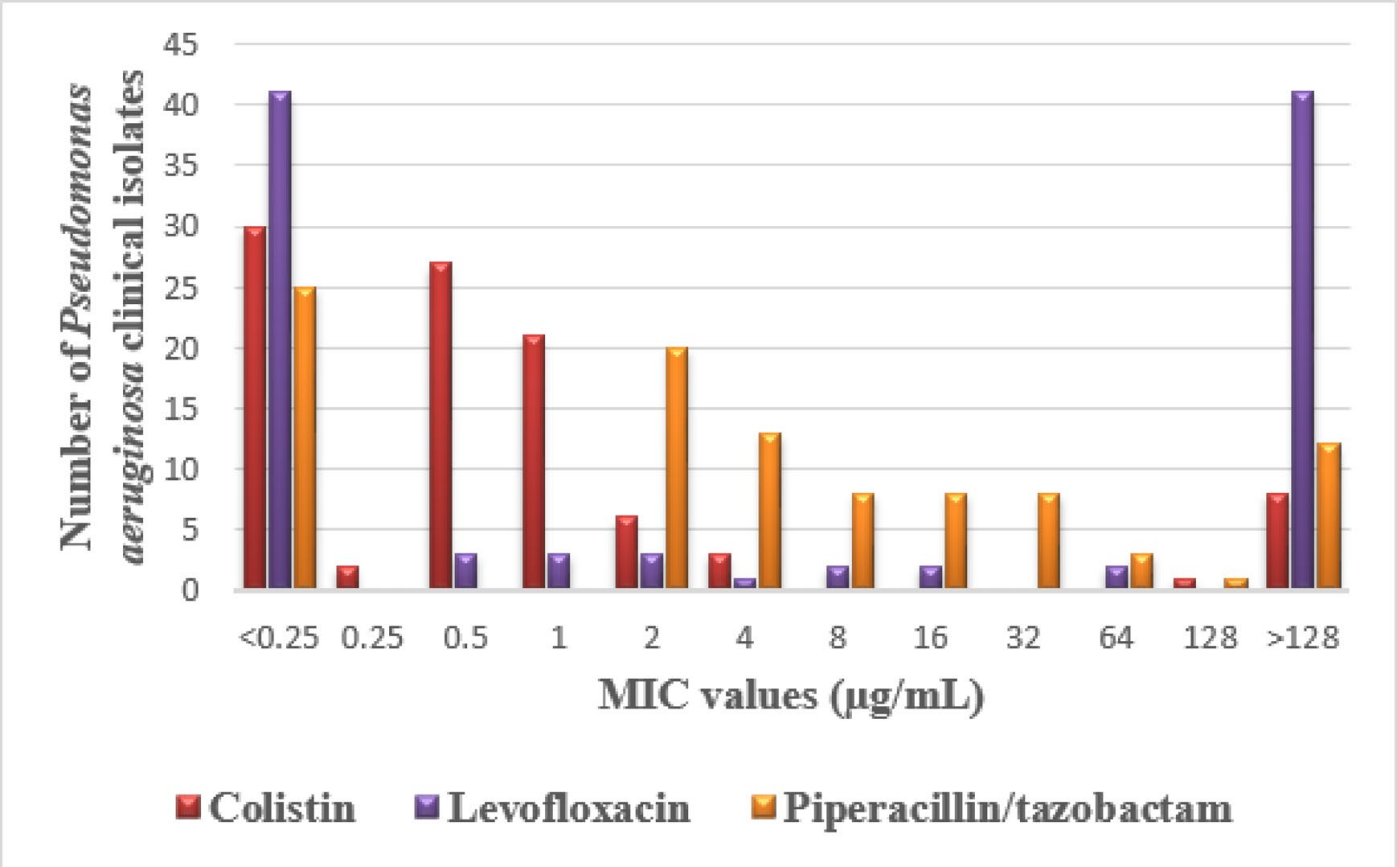
The minimum inhibitory concentration (MIC) values of the tested antimicrobial agents against the *Pseudomonas aeruginosa* clinical isolates.

Results derived from the MIC test showed that the majority of *Burkholderia* species, including the Bcc isolates, were susceptible to levofloxacin, with 9 (75%) susceptible isolates, then piperacillin/tazobactam, with 7 (58.3%) susceptible isolates, while colistin had only 2 (16.6%) susceptible isolates. On the other hand, the majority of *P. aeruginosa* isolates were susceptible to colistin, with 80 (81.6%) susceptible isolates, followed by piperacillin/tazobactam, with 74 (75.5%) susceptible isolates, then levofloxacin, with 47 (47.9%) susceptible isolates. The MIC values for these antimicrobial agents were illustrated in Figs. 6 and 7.

#### Pattern of the antimicrobial susceptibility

Considering all the tested antimicrobial agents (using Kirby-Bauer and broth microdilution methods), the majority of *Burkholderia* species, including the Bcc isolates, were susceptible to cotrimoxazole, with 11 (91.6%) susceptible isolates, followed by minocycline and meropenem, with 10 (83.3%) susceptible isolates, levofloxacin, with 9 (75%) susceptible isolates, piperacillin/tazobactam, with 7 (58.3%) susceptible isolates, gentamicin, with 4 (33.3%) susceptible isolates. None of the *Burkholderia* isolates were susceptible to ceftazidime, followed by colistin as the second least effective antimicrobial agent, with only 2 (16.6%) susceptible isolates.

the majority of *P. aeruginosa* isolates were susceptible to colistin, with 80 (81.6%) susceptible isolates followed by piperacillin /tazobactam, with 74 (75.5%) susceptible isolates, meropenem, with 59 (60.2%) susceptible isolates, gentamicin, with 55 (56.1%) susceptible isolates, ceftazidime, with 50 (51%) susceptible isolates, levofloxacin, with 47 (47.9%) susceptible isolates, and cotrimoxazole, with 46 (46.9%) susceptible isolates while minocycline has the least activity, with only 6 (6.12%) susceptible isolates.

A statistically significant difference in results was observed between the *Burkholderia* species and *P. aeruginosa* isolates tested for colistin, cotrimoxazole, ceftazidime, and minocycline, with a p-value less than 0.05. The majority of *P. aeruginosa* isolates were susceptible to colistin and ceftazidime, while *Burkholderia* isolates were more susceptible to cotrimoxazole and minocycline.

### Detection of multidrug-resistant bacteria

The results showed that out of the 110 clinical isolates, 45 (40.9%) isolates were resistant to 3 or more classes of antimicrobial agents and so can be considered MDR, while 65 (59.1%) isolates were non-MDR. Forty-one MDR isolates were *P. aeruginosa*, representing 37.3% out of the total 110 clinical isolates, while representing 41.8% out of the *P. aeruginosa* isolates. For *Burkholderia* species, 4 isolates were MDR, representing 3.6% out of the total 110 clinical isolates tested, while representing 33.3% out of the total *Burkholderia* isolates.

A heatmap represents phenotypic, genotypic, and antimicrobial susceptibility testing results for each clinical isolate tested in addition to the standard strains was provided as Supplementary Table 2.

### Determination of the effect of antimicrobial combinations

#### Double disc synergy assay

Synergy was indicated by bridging the zone of inhibition with the antimicrobial combinations` gentamicin and meropenem, in addition to the meropenem and cotrimoxazole combination against both BAA/245 and PAO1. Double disk synergy test results are illustrated in Supplementary Fig. 2.

#### Checkerboard assay result

Synergism and partial synergism were observed with the CN + MRP combination, while only partial synergism was observed for the NALC + MRP combination and NALC + LVX combination. On the other hand, an indifference effect was observed for the LVX + MRP combination and the COT + MRP combination against both standards. Partial synergism was observed for the TZP + CN combination against PAO1. However, only the indifference effect was observed against BAA/245. None of the combinations showed any antagonistic interaction. Concentrations at which synergism and partial synergism were observed are illustrated in Table 1.

**Table 1:** Concentrations of antimicrobial agents at which synergism and partial synergism were observed.

Antimicrobial Combinations	Standard strain	Synergism (µg/mL)	Partial synergism (µg/mL)
**CN + MRP**	BAA/245	64 + 4	8 + 8
		256 + 1	1024 + 1
	PAO1	0.25 + 1	0.125 + 2
			0.5 + 1
**NALC + MRP**	BAA/245	ND	3125 + 1
	PAO1	ND	780 + 2
			3125 + 0.5
**NALC + LVX**	BAA/245	ND	3125 + 1
	PAO1	ND	1562 + 0.125
			3125 + 0.06
**TZP + CN**	PAO1	1 + 0.125	2 + 0.06
		1 + 0.25	0.5 + 0.5

*CN: gentamicin, MRP: meropenem, NALC: N-Acetyl-L-Cysteine, LVX: levofloxacin, TZP: piperacillin/tazobactam, BAA/245: the standard strain of Bcc, PAO1: the standard strain of *P. aeruginosa*, ND: not detected.

#### Dual antimicrobial combination result using multiple combination bactericidal test

Combinations of two antimicrobial agents against 53 clinical isolates were tested; 12 of them were *Burkholderia* species, and 41 were MDR *P. aeruginosa* clinical isolates. The dual antimicrobial combinations tested against the clinical isolates were (CN + MRP, CN + TZP, NALC + MRP, and NALC + LVX). The selected antimicrobial agents used in these combinations were the most effective antimicrobials that were tested previously on both standards (BAA/245 and PAO1). The combinations were considered synergistic if bactericidal activity of 99.9% killing was achieved.

**Fig. 8:**
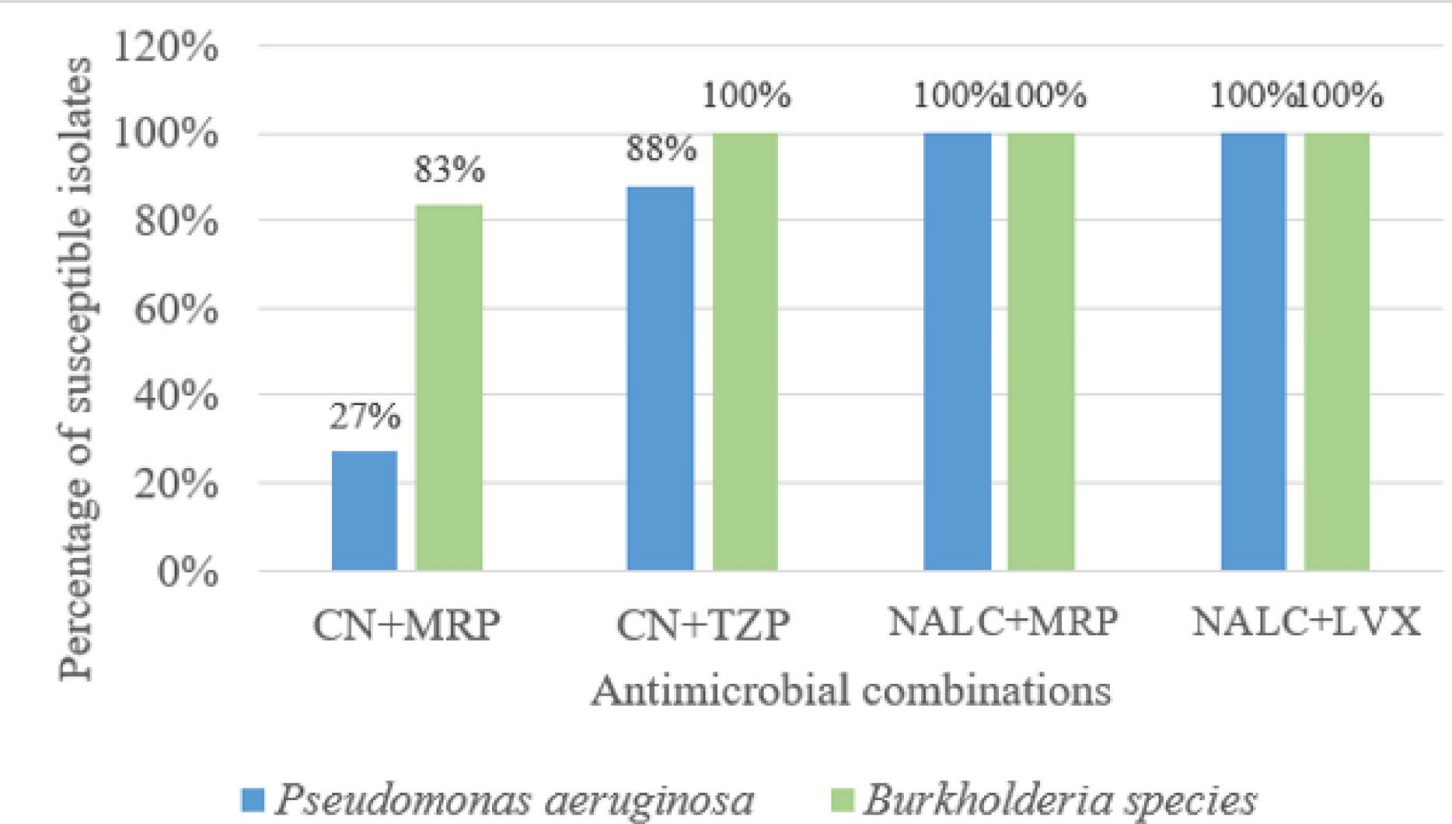
**The effect of dual antimicrobial combinations against tested clinical isolates**. *CN: gentamicin, MRP: meropenem, TZP: piperacillin/tazobactam, NALC: N-Acetyl-L-Cysteine, LVX: levofloxacin. **Note that**: The percentage of susceptible isolates for either Bcc or *P. aeruginosa* is correlated to the number of isolates tested for that microorganism.

The combination of CN + MRP showed a synergistic effect against 21/53 (39.6%) isolates (11/21 *P. aeruginosa* and 10/21 *Burkholderia* species) while the CN + TZP combination showed a synergistic effect against 48/53 (90.5%) isolates (36/48 *P. aeruginosa* and 12/48 *Burkholderia* species). On the other hand, NALC + MRP and NALC + LVX combinations showed synergistic effect against all the tested isolates (*n* = 53) **(**Fig. 8**)**.

A statistically significant difference in results was observed between the *Burkholderia* species and MDR *P. aeruginosa* isolates tested for CN + MRP combination, which was more effective against *Burkholderia* species isolates, while CN + TZP was effective on both microorganisms, so there was no statistically significant difference in results between *Burkholderia* species and MDR *P. aeruginosa* isolates. A comparison of the susceptibility for the dual combinations against *Burkholderia* species and MDR *P. aeruginosa* isolates tested, in addition to the p-value calculated for each combination, was illustrated in Table 2.

**Table 2:** Comparison of the susceptibility for the dual combinations tested.

Dual antimicrobial combinations	Susceptible No. (%)		Not susceptible No. (%)		*p*-Value
	*Burkholderia* species (*N* = 12)	*P. aeruginosa* (*N* = 41)	*Burkholderia* species (*N* = 12)	*P. aeruginosa* (*N* = 41)	
MRP + CN	10 (83.3%)	11 (26.8%)	2 (16.7%)	30 (73.2%)	0.001
**TZP + CN**	**12 (100%)**	**36 (87.8%)**	**0 (0%)**	**5 (12.2%)**	**0.577**
**NALC + MRP**	**12 (100%)**	**41 (100%)**	**0 (0%)**	**0 (0%)**	-*
**NALC + LVX**	**12 (100%)**	**41 (100%)**	**0 (0%)**	**0 (0%)**	-*

* MRP: meropenem, TZP: piperacillin/tazobactam, CN: gentamicin, NALC: N-Acetyl-L-Cysteine, LVX: levofloxacin. **Note that**: Dual combinations were tested against *Burkholderia* species and multidrug-resistant *Pseudomonas aeruginosa* isolates. No statistics were computed for (NALC + MRP) and (NALC + LVX) because both had constant values.

#### Triple antimicrobial combination result using multiple combination bactericidal test

**Fig. 9:**
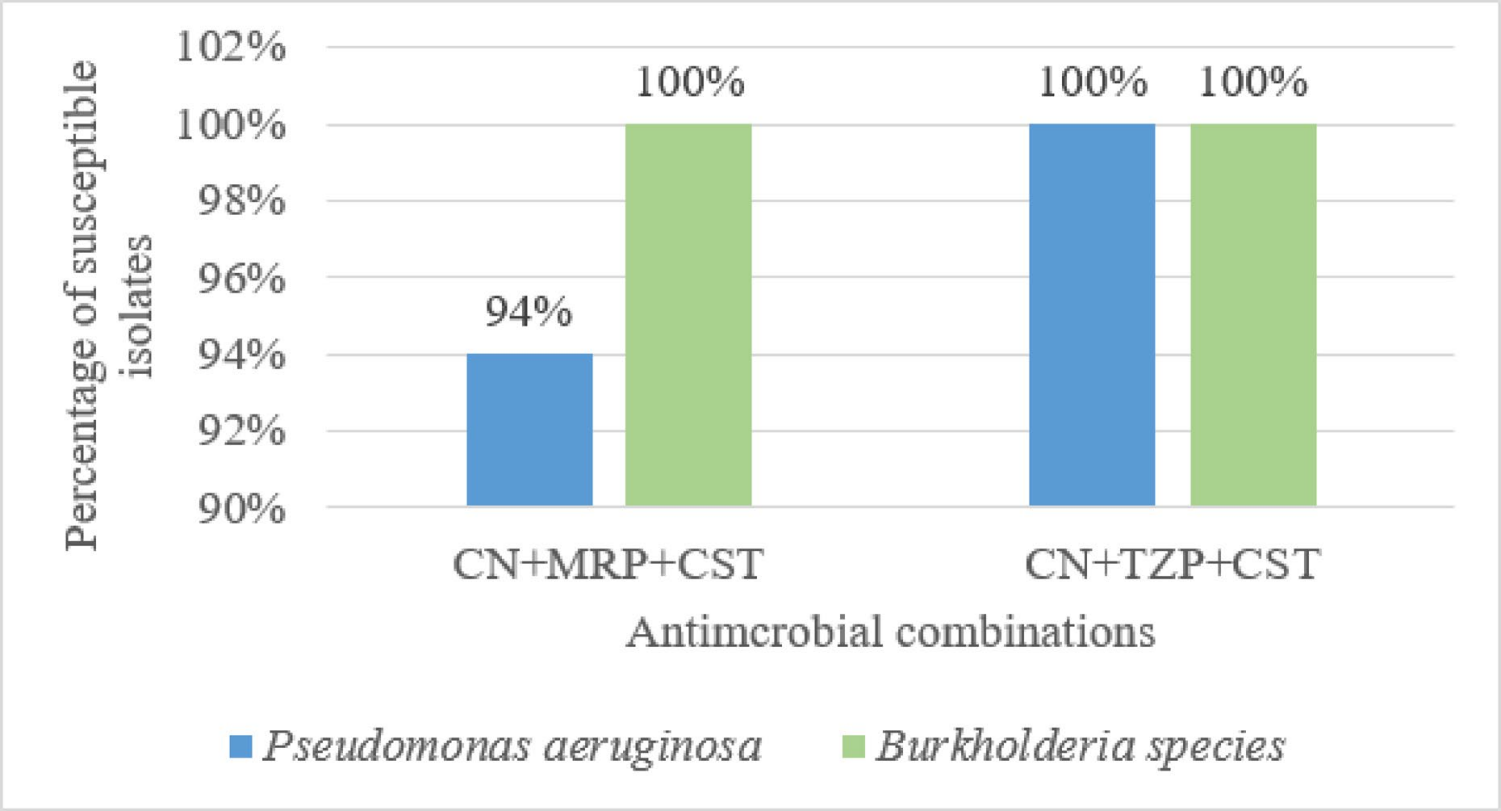
The effect of the triple antimicrobial combinations against tested clinical isolates. *CN: gentamicin, MRP: meropenem, TZP: piperacillin/tazobactam, CST: colistin. Note that: The percentage of susceptible isolates for either Bcc or *P. aeruginosa* is correlated to the number of isolates tested for that microorganism.

Combinations of three antimicrobial agents were tested against clinical isolates that were resistant to the dual antimicrobial combinations. The triple combinations included in the study were (CN + MRP + CST) combination which was tested against 32 clinical isolates resistant to the dual combination (CN + MRP), 2 of them were *Burkholderia* species and 30 were MDR *P. aeruginosa* clinical isolates, in addition to (CN + TZP + CST) combination which was tested against 5 clinical isolates resistant to the dual combination (CN + TZP). The first combination (CN + MRP + CST) showed synergistic effect against 30/32 (93.75%) isolates with only 2 *P. aeruginosa* isolates resistant, while the second combination (CN + TZP + CST) showed synergistic effect against all the tested isolates (*n* = 5) (Fig. 9). The susceptibility of the isolates for each single antimicrobial agent tested in the combinations as well as the effect of the dual and triple combinations were illustrated in Supplementary Table 3.

## Discussion

Both Bcc and *P. aeruginosa* are opportunistic pathogens that are isolated from different sources^24^. Bacteria of Bcc need to be correctly identified from *P. aeruginosa* because of its markedly different susceptibility pattern. Also, it has intrinsic resistance to several antimicrobials frequently used to treat *Pseudomonas* infections, so accurate Bcc identification is essential^2,8,17^. Otherwise, targeting both pathogens in the treatment strategy would ensure timely and effective management until precise microbiological results are available.

In our study, Phenotypic differentiation was carried out using conventional phenotypic methods in addition to the automated VITEK 2 system. Conventional phenotypic methods showed unreliability in the differentiation between these two pathogens, while the automated method showed a higher discrimination ability.

In comparison with other studies, Moehario et al. reported that VITEK 2 achieved an accuracy of 96.77% at the genus level and 90.32% at the species level concerning the correct identification of *P. aeruginosa*^43^. Zbinden et al.. reported that almost all identifications performed using the VITEK 2 system in their study were correct for *Stenotrophomonas maltophilia*,* Achromobacter xylosoxidans*,* P. aeruginosa*, and Bcc^44^. On the other hand, Devanga and Veeraraghavan reported that conventional biochemical methods cannot distinguish between Bcc and non-Bcc, while automated phenotypic identification using VITEK 2 is only reliable in genus-level identification, and species-level identification cannot be achieved due to the high similarity of biochemical results between species^10^. Moreover, Gautam et al. reported that species such as *Pandoraea* species and *Ralstonia picketti* (non-*Burkholderia* beta proteobacteria) could be mistakenly identified as Bcc, while some Bcc could be mistaken for *P. aeruginosa*^4^. This suggests that isolates identified by these systems as Bcc need to be validated.

Susceptibility testing has been recognized as a screening strategy for the identification of microorganisms^45^. Testing for intrinsic resistance in our study revealed that only colistin showed different susceptibility in results between standard *B. cenocepacia* and *P. aeruginosa*, so all the colistin-resistant clinical isolates were suspected to be Bcc. Our findings were consistent with the study of Meena et al., which reported that Bcc was preliminarily isolated as Gram-negative, oxidase-positive, non-lactose fermenting bacilli, which were resistant to colistin and aminoglycosides but susceptible to cotrimoxazole and were further confirmed by the VITEK 2 system^46^. Also, Somprasong et al. used susceptibility testing as a screening strategy in their study. They reported that testing for susceptibility to amoxicillin-clavulanic acid, together with resistance to gentamicin and colistin, is one of the agreed criteria for identifying *B. pseudomallei* in specimens from patients and the environment^45^.

Our study confirmed 4 clinical isolates as Bcc using the PCR amplification of the *recA* gene with the aid of specific primers for Bcc^14,15^ while for the species-level identification, sequence analysis of the *recA* gene showed high effectiveness. Studies reported that PCR amplification of the *recA* gene has a higher specificity for identifying Bcc bacteria than 16 S and 23 S rRNA sequencing^14^. The sequence analysis of the 16 S rRNA gene is widely used for the identification of bacteria. However, for Bcc, it is only able to recognize the genus and differentiate it from other related genera without recognizing the exact species^10^.

The sequence analysis of the *recA* gene amplified products revealed that all the 4 tested sequences in our study were *B. cenocepacia* (member of the Bcc), a highly virulent species^47^ which is included in the 5 Bcc species that were believed to be able to spread through aerosol droplets. This implies that these bacteria can spread quickly in nosocomial settings within vulnerable patients by means of direct as well as indirect contact with other patients who are infected^3,47^.

The *recA* gene is a small fragment out of the MLST loci that can provide species-level identification similar to MLST loci. Sequencing of the *recA* gene provides an inexpensive and rapid tool for adequate species-level identification^48^.

However, the sample size can be considered as a limitation in our study, it reflects the actual clinical prevalence patterns observed, where *P. aeruginosa* was a more common pathogen compared to *Burkholderia*. Future multicenter collaborations or extended surveillance periods could help improve the reliability of observed data.

Newer identification methods as MALDI-TOF MS have gained interest in the accurate identification of bacteria recently, but for Bcc isolates, it showed deficiencies in differentiating the different members to the species level based on different studies^10,49^.

Decreased susceptibility of the *Burkholderia* isolates was observed with colistin, which was expected due to intrinsic resistance^34^. On the other hand, there was high susceptibility to cotrimoxazole, followed by minocycline and meropenem. A finding that was partially consistent with the results of Lee et al. study, which reported high susceptibility rates for cotrimoxazole, piperacillin/tazobactam, and ceftazidime against isolates of Bcc^36^. Another study conducted by Omar et al.. reported that Bcc was most susceptible to ceftazidime and meropenem, followed by tobramycin, and they were found to have 100% resistance to cotrimoxazole and ciprofloxacin^12^. Our study had proved the high susceptibility of *Burkholderia* isolates to cotrimoxazole and meropenem, but unlike what has been mentioned in the two previous studies, there was 100% resistance of *Burkholderia* isolates against ceftazidime. In contrast to Omar et al.. cotrimoxazole was the most effective antimicrobial agent tested, a result that was supported by the other previously mentioned reference. These variable findings could be explained by the organism’s susceptibility to cotrimoxazole and antipseudomonal beta-lactams during its initial isolation. However, with antimicrobial pressure, resistance grows quickly, and clinicians have to manage infections in which the organism is resistant to available antimicrobial agents^4^.

On the other hand, the majority of *P. aeruginosa* isolates were more susceptible to colistin; this result is consistent with the study of Bae et al., which described colistin as the most active agent against MDR Gram-negative pathogens in-vitro^40^, indicated by the fact that it is the antimicrobial agent of last resort for treating MDR bacteria^34,50^. In contrast, *Burkholderia* isolates had much less susceptibility percentage to colistin due to intrinsic resistance. A statistically significant difference can be observed in susceptibility results for this antibiotic between *Burkholderia* species and *P. aeruginosa* isolates.

Isolates of *P. aeruginosa* showed low susceptibility percentage towards minocycline and cotrimoxazole, which can be attributed to the fact that *P. aeruginosa* is intrinsically resistant to both of them^34^ unlike *Burkholderia* isolates, which had the highest susceptibility percentage to cotrimoxazole, followed by minocycline. A statistically significant difference was observed in susceptibility results for cotrimoxazole and minocycline between *Burkholderia* species and *P. aeruginosa* isolates.

These differences in susceptibility results between *P. aeruginosa* and Bcc support the fact of having a contrasting susceptibility pattern, which highlights the value of proper identification^4,12,22^. We further investigated different therapeutic perspectives that can have the ability to disrupt the multidrug, intrinsic, high-level resistance of Bcc, together with the notorious *P. aeruginosa* pathogen, aiming to overcome the effect of the misidentification possibility that had been reported in several other studies^9–11^ and proved in our study, especially in developing countries, where the molecular techniques are not regularly employed^18^.

The susceptibility of BAA/245 and PAO1 was tested against NALC, and both were inhibited at the same concentration of 6250 µg/mL. A result that was very close to that reported in the Young et al.. study, where the MIC of NALC against PAO1, the same *P. aeruginosa* standard strain used in our study, was 5000 µg/mL^51^. On the other hand, Pollini et al.. reported that the MICs of NALC against 19 different Bcc strains were in the range of 16,000 to 32,000 µg/mL^24^. The NALC antibacterial and antibiofilm activities have been reported at variable concentrations, although these have generally been higher than those achieved via systemic administration routes (intravenous, oral, or intramuscular), which can achieve plasma concentrations in the range of 200–1200 µg/mL^52^. However, NALC can also be used by nebulization or direct instillation to obtain the higher concentrations required for the antibacterial and antibiofilm activities at the infection site^24^.

The currently recorded synergism between beta-lactams and gentamicin could be justified by that the combination of beta-lactam (meropenem or piperacillin/tazobactam) and aminoglycoside (gentamicin) supports different mechanisms of bacterial killing^53^. It is commonly recognized that the most effective treatment for severe *P. aeruginosa* infections is a beta-lactam plus an aminoglycoside combination^54^.

A study by Aksoy et al.. reported that the addition of NALC significantly enhanced the meropenem efficacy against tested clinical isolates, including *P. aeruginosa*^55^ while for the NALC and levofloxacin combination, Aiyer et al.. reported that synergy of ciprofloxacin (another member of fluoroquinolones) and NALC was observed for *B. cenocepacia* strains E5328 and E5452^26^ and in another study, it was reported that NALC with ciprofloxacin combination is both bactericidal and disruptive, besides providing an alternative approach that can reduce the burden of antibiotics targeting difficult-to-treat infections^56^. Conversely, Landini et al.. investigated the efficacy of NALC both alone and in combination with beta-lactams against a variety of Gram-positive and Gram-negative bacteria and discovered that in the majority of strains tested, NALC MICs were higher than 16 mg/mL. The addition of NALC to antibiotics did not result in any detectable synergistic interaction. Furthermore, NALC adversely affected the carbapenem activity, with imipenem being more significantly affected than ertapenem and meropenem^57^.

Although antimicrobial synergy seems to be well-established for beta-lactams and aminoglycoside combinations, data on synergistic activity have also appeared for beta-lactams and fluoroquinolones combinations. As an effective alternative to aminoglycosides, fluoroquinolones are safer and have been used extensively^58^ but unfortunately, on testing the effect of meropenem and levofloxacin combination in our study against both standards, no synergism or partial synergism were detected, which is consistent with what`s mentioned by Erdem et al.. that levofloxacin and imipenem or meropenem were not synergistic against any *P. aeruginosa* strain tested in their study, and none of the combinations showed an antagonistic effect^59^. For the meropenem and cotrimoxazole combination, only an indifference effect was observed against both standards. Similarly, Dales et al.. reported that the combination of meropenem and cotrimoxazole had the lowest percentage of inhibition (20%, 12%) against the tested *P. aeruginosa* and Bcc isolates, respectively^60^.

Triple combinations are emerging as a tool to overtake multidrug resistance^61^. Despite its lack of activity against Bcc and nephrotoxicity, colistin was added to the previously tested dual combinations against some isolates that showed resistance, owing to the reported ability of colistin to permeabilize the cell envelope of Gram-negative bacteria to other antibiotics, even when used in very low concentrations^62^.

The following triple combinations were tested in our study: MRP + CN + CST in addition to TZP + CN + CST, and results revealed that both of the triple combinations tested had a synergistic and bactericidal effect on both the standards and clinical isolates tested, leaving only 2 *P. aeruginosa* isolates still resistant to the MRP + CN + CST combination.

In comparison with other studies, Dales et al.. reported that the triple antimicrobial combinations that inhibited the growth of most *P. aeruginosa* and Bcc isolates tested in their study were tobramycin in high-dose, which can be achieved by inhalation, added to meropenem plus a third intravenous antimicrobial agent (piperacillin/tazobactam, amikacin, ceftazidime, or cotrimoxazole)^60^. Moreover, El-halfawy et al.. reported that ceftazidime and moxifloxacin combination inhibited 16 clinical isolates of Bcc at physiologically achievable concentrations. Adding a low concentration of colistin improved the combination efficacy^62^ and it was also reported that the combination of doripenem (another member of carbapenems) added to polymyxin B plus rifampin at 1/4 MICs for each antimicrobial agent had bactericidal activity against all *P. aeruginosa* isolates tested^63^.

However, treatment with antimicrobial combinations had significant concerns on patient outcomes and antimicrobial stewardship, observational studies conducted in the past decade indicated that combination of two or more antimicrobial agents as a treatment of MDR infections had better patient outcomes in comparison with monotherapy, at least in critically ill patients, as stated by an international cross-sectional survey performed by ESCMID in large hospitals. They reported that treatment with antimicrobial combinations was conducted in at least 114/115 (99.13%) hospitals^64^. Another review conducted by our team reported that Bcc infections had heterogeneous treatment strategies. However, the majority consisted of a combination of nebulized, oral, and intravenous antimicrobial agents^65^.

Even though various studies suggested using antimicrobial combinations to treat MDR infections, easily accessible patient medical records and current data on the local microbiological epidemiology are still essential for establishing the baseline threat of infections and guiding decisions regarding empirical treatment to prevent both overtreatment and undertreatment. Additionally, rapid and efficient diagnosis and laboratory processes are crucial to narrow the antimicrobial spectrum for purposes of de-escalation and aligning with the principles of antimicrobial stewardship^66^.

## Conclusion

Accurate identification is essential for early diagnosis of hospital-acquired infections and administration of the proper antimicrobial treatment. The differences in the previously illustrated susceptibility test results between *P. aeruginosa* and Bcc support the fact of having contrast susceptibility pattern, which highlight the value of proper identification and differentiation in addition to targeting treatment options that can eradicate both microorganisms. The results of the dual and triple combinations tests demonstrated the possibility that NALC + MRP and NALC + LVX combinations may help managing both *P. aeruginosa* and Bcc infections. These combinations deserve to be further studied for possible clinical applications, particularly for inhaled formulations that would enable the high NALC concentration to be reached at the infection site in cases with pulmonary infections while minimizing systemic toxicity. Furthermore, delivery systems based on nanotechnology may enable delivering even higher dosages of medication to the infection site. For conventionally used antibiotics, isolates of both pathogens were more susceptible to the CN + TZP combination at concentrations that can be readily used in patients, and only a small percentage of isolates exhibited resistance that was totally recovered by adding colistin, also at physiologically achievable concentrations, and that triple combination had a remarkable bactericidal activity. Although the CN + MRP combination had the lowest bactericidal activity with 32 (60.3%) isolates resistant, adding also colistin greatly enhanced the activity, leaving only 2 (3.7%) isolates still resistant.

## Supplementary Information

Below is the link to the electronic supplementary material.

Supplementary Material 1

Supplementary Material 2

## Data Availability

The datasets generated or analyzed during the current study are available in the NCBI Sequence Read Archive repository, https://www.ncbi.nlm.nih.gov/sra/?term=PRJNA1295989. Raw data of the recA gene sequences are available under BioProject accession number: PRJNA1295989 for the four sequenced isolates, Bur5, Bur8, Bur9, and Bur10, with BioSample accessions SAMN50194177, SAMN50194178, SAMN50194179, and SAMN50194180, respectively.

## Abbreviations

BAA/245: Standard Burkholderia cenocepacia
Bcc: Burkholderia cepacia complex
BCSA: Burkholderia cepacia selective agar
CAZ: Ceftazidime
CF: Cystic fibrosis
CN: Gentamicin
COT: Cotrimoxazole
CST: Colistin
FIC: Fractional inhibitory concentration
ICU: Intensive care unit
IZDs: Inhibition zone diameters
LVX: Levofloxacin
MCBT: Multiple combination bactericidal test
MDR: Multi-drug resistance
MI: Minocycline
MIC: Minimum inhibitory concentration
MRP: Meropenem
NALC: N-Acetyl-L-Cysteine
PAO1: Standard Pseudomonas aeruginosa
PCR: Polymerase chain reaction
TZP: Piperacillin/tazobactam
