# Supplementary material for: Effective therapeutic strategies against Pseudomonas aeruginosa and Burkholderia Cepacia complex infections
Source: Sci Rep. 2025 Nov 23;15:41462. doi: 10.1038/s41598-025-26712-8 (PMC12644862; doi:10.1038/s41598-025-26712-8)
Supplement: Supplementary file 2 — Supplementary Material 2 [file 41598_2025_26712_MOESM2_ESM.docx]

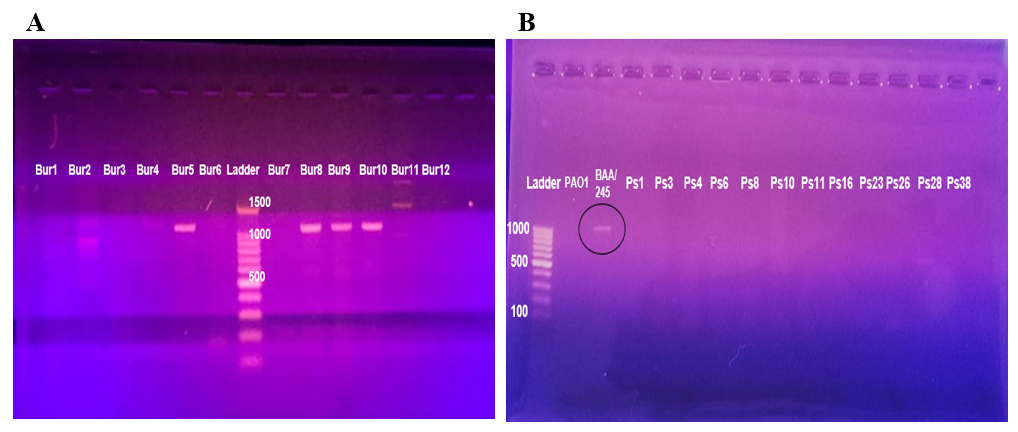


**b**

**a**

Supplementary Figure 1: Ethidium bromide-stained gel electrophoresis for the tested clinical isolates` PCR products.

*a. Only 4 isolates (Bur5, Bur8, Bur9, and Bur10) gave bands of the expected size of the *recA* gene at 1040 base pairs; b. Only BAA/245 gave a band of the expected size of the *recA* gene at 1040 base pairs.

**b**

**a**

**
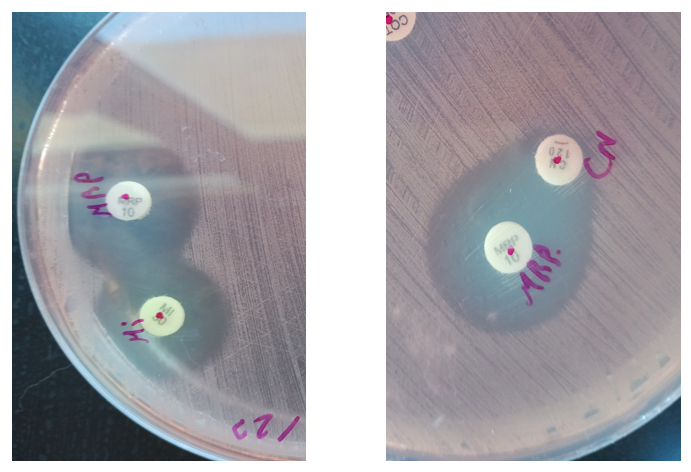
**

**Supplementary Figure 2:** **Double disk synergy test result.**

***a.** Indifference/additive (no effect on the zone of inhibition); **b.** Synergy (bridging of the zone of inhibition).
